# Parasitic worms and inflammatory diseases

**DOI:** 10.1111/j.1365-3024.2006.00879.x

**Published:** 2006-10

**Authors:** P ZACCONE, Z FEHERVARI, J M PHILLIPS, D W DUNNE, A COOKE

**Affiliations:** Department of Pathology Tennis Court Road, Cambridge, UK

**Keywords:** helminth, Hygiene Hypothesis, immunomodulation, *Schistosoma mansoni*, type 1 diabetes

## Abstract

The debate on whether infection precipitates or prevents autoimmunity remains a contentious one. Recently the suggestion that some unknown microbe can be at the origin of some chronic inflammatory diseases has been countered by accumulating evidence that decreasing infection rates might have an important role to play in the rising prevalence of autoimmune disorders. The ‘Hygiene Hypothesis’ was initially postulated to explain the inverse correlation between the incidence of infections and the rise of allergic diseases, particularly in the developed world. Latterly, the Hygiene Hypothesis has been extended to also incorporate autoimmune diseases in general. Amongst the various infectious agents, a particular emphasis has been put on the interaction between parasitic worms and humans. Worm parasites have co-evolved with the mammalian immune system for many millions of years and during this time, they have developed extremely effective strategies to modulate and evade host defences and so maintain their evolutionary fitness. It is therefore reasonable to conclude that the human immune system has been shaped by its relationship with parasitic worms and this may be a necessary requirement for maintaining our immunological health. Fully understanding this relationship may lead to novel and effective treatments for a host of deleterious inflammatory reactions.

## INTRODUCTION

The last three decades have witnessed a dramatic increase in the incidence of autoimmune inflammatory diseases in developed countries, including type 1 diabetes (T1D), multiple sclerosis (MS), rheumatoid arthritis (RA), and Crohn's disease, to name but a few (1,2). Autoimmune disease is characterized by an immune-mediated attack on a target organ that it is no longer recognized by the immune system as self. Autoimmune pathology can be caused by both antibody and cell-mediated components. Predisposition to autoimmunity is under polygenic control, but studies on identical (monozygotic) twins demonstrate that environmental factors might be equally important (3,4). The rising trend in autoimmune diseases looks set to continue and the projected incidences over the next 30 years are potentially catastrophic. The countries that have seen the most pronounced rise in autoimmunity have over the same period seen tremendous improvements in sanitation and socioeconomic status. Moreover, the steady migration from rural to urban areas has dramatically reduced childhood exposure to infectious organisms. Rapid anthropogenic transformation of the environment and life style has not allowed time for the human immune system to adjust to these changes. The very characteristics of the immune system that had previously been so advantageous for combating infections might now be the principal contributing factor for the increasing prevalence of autoimmune disease. Improvements in living conditions and the reduced exposure to childhood infections in particular, have been suggested to contribute to the increase in atopy and autoimmunity (5). This so-called *Hygiene Hypothesis* has, in recent years, attracted interest and controversy in equal measure. Epidemiological data from the World Health Organization (WHO) largely support the hypothesis, indicating that autoimmune inflammatory diseases like T1D and MS are extremely rare in most African and Asian populations, yet increase conspicuously when these same populations migrate to a modern setting (6,7). In this piece we will review the evidence for the Hygiene Hypothesis particularly with regard to parasitic worm (helminth) infections and consider any potential therapeutic avenues that it may prescribe.

## INFECTIOUS DISEASE, AUTOIMMUNITY, AND THE HYGIENE HYPOTHESIS

The Hygiene Hypothesis suggests that parasites and microbes have been important for shaping and tuning the evolution of the human immune system (8). According to this hypothesis, the immune system is in a state of preparedness, primed to repel the pathogen assaults that characterized the lot of humanity for most of its existence. In developed countries industrialization has strongly contributed to human migration from rural areas to the cities. One of the consequences of resettlement has been the removal of people from the pathogen-replete ecosystems in which their immune systems had adapted since prehistory. Sanitation, and access to clean food and water became a common life standard for most individuals in the developed world. Additionally, following the Second World War the use of antibiotics became commonplace, dramatically altering exposure to bacterial pathogens. The fact that infections were no longer prevalent has led to the emergence of autoimmune inflammatory diseases. This suggests that parasites, if not actually preventing autoimmunity *per se*, at least divert the immune system to the more productive cause of limiting tissue pathology. Parasites themselves wield an astonishing array of mechanisms to evade the ravages of the host's immune system and in so doing ameliorate the more self-destructive aspects of a response.

**Figure 1:**
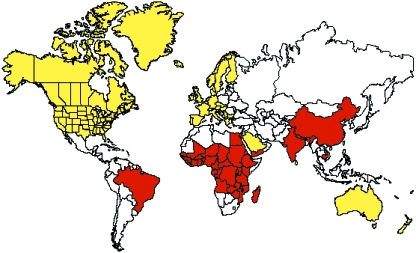
Inverse correlation between Type 1 Diabetes (T1D) and ‘neglected infectious diseases’. Red delineates areas which harbour six or more of the low mortality neglected diseases (filariasis, leprosy, onchocerciasis, schistosomiasis, soil-transmitted helminths, and trachoma). Yellow delineates areas where there are relatively high incidences of T1D (> 8 per 100 000/year). Non coloured areas delineate where T1D < 8 per 100 000/year and where the ‘neglected diseases’ are not endemic.

Industrialized countries are indeed experiencing an increase in autoimmune diseases. A very different picture is present in developing countries. Because of limited economic resources, health aid organizations tend to focus more on the three so-called ‘Big Killer Diseases’; HIV, malaria and tuberculosis. As a consequence, six neglected infectious diseases with low mortality rates such as filariasis, leprosy, onchocerciasis, schistosomiasis, soil-transmitted helminths, and trachoma, are still widespread yet autoimmune inflammatory diseases are virtually absent (WHO and International Diabetes Federation databases) (see Figure 1). Loss of parasite colonization in those individuals living in developed countries has had a unique impact on our immune response and, together with genetic predisposition, is probably the pre-eminent factor contributing to the development of autoimmune disease (9–12).

T1D occurs equally among males and females and is more common in whites than in non-whites. Data from the IDF (International Diabetes Federation) database indicate that T1D is rare in most African and Asian populations. Conversely, some northern European (Finland and Sweden) and northern American countries, have high rates of T1D. Over a million people worldwide have MS and this incidence also appears to be increasing. Onset of symptoms typically occurs between the ages of 15 and 40 years, with a peak incidence in people in their 20s and 30s, and women are affected twice as often as men. MS occurs worldwide but is most common in Caucasian people of northern European origin. It is extremely rare among Asians and Africans (13). Crohn's disease also occurs most frequently among North Europeans and North Americans. Although the disorder can begin at any age, its onset principally occurs between 15 and 30 years of age. There appears to be a familial aggregation of patients with Crohn's disease such that 20–30% of patients with Crohn's disease have a family history of inflammatory bowel disease.

## T1D AS AN EXAMPLE OF AN INFLAMMATORY AUTOIMMUNE DISEASE

T1D is an autoimmune condition characterized by a progressive cellular infiltration of the pancreas resulting in the destruction of insulin-producing cells. Since insulin regulates glucose uptake into cells from the circulation, its deficiency is responsible for glucose accumulation in the blood and ensuing cell starvation (hyperglycaemia, coma, etc.). T1D was considered a death sentence until the early 1920s, when pancreatic extracts were used to correct hyperglycaemia (14). This discovery led to the availability of an effective treatment – insulin injections – and the first clinical patient was treated in 1922. Despite the substantial technological improvement for monitoring glycaemia, relatively little progress has been made in terms of therapy; to date, insulin injection remains the only dependable treatment.

T1D is an autoimmune polygenic disorder, with numerous gene loci contributing to susceptibility. Historically, the first genes associated with T1D were the Human Leukocyte Antigens (HLA) on chromosome 6, in particular the DR and DQ class II regions (15,16). There remains controversy about the relative contributions of DR and DQ on T1D susceptibility with some studies supporting a stronger association for the DQ locus and a secondary role for DR (17). Recently, other genes outside the HLA complex have been associated with predisposition to T1D, including cytotoxic T lymphocyte associated antigen 4 (CTLA-4) and lymphoid tyrosine phosphatase (PTPN22) (18).

As mentioned above, genetic components affect the propensity for T1D but the environment appears to play a fundamental role in regulating the onset of the disease. Many different intercepting factors must be taken into account. Within the Caucasian population the incidence of T1D varies between nations. For example Scandinavian countries have the highest incidence of T1D in Europe, whereas relatively under-developed countries like Albania and Romania have some of the lowest (see Table 1). However, these statistics need to be considered in the context of genetics, i.e. the relatively limited genetic diversity seen in Scandinavia (particularly Finland) overlaying and possibly synergizing with the effects of a hygienic environment. Interestingly in Europe, countries with a more agriculture-based economy have lower incidences of T1D (19). This suggests that exposure of the population to a diet containing fewer processed foods and more direct contact with animal-transmitted pathogens such as *Salmonella* could be a relevant factor in preventing T1D. The North–South gradient also seems to play a role in diabetes incidence. Indeed in Southern European countries the lower socio-economic status and higher temperatures might predispose the inhabitants to infections and contribute to the lower frequency of T1D. Two of the largest islands in the Mediterranean, Sicily and Sardinia, present an interesting contrast in T1D incidence and the effects of genetics. These islands are located at similar latitudes and bio-geographical zones, yet Sicily has a low incidence of T1D whereas Sardinia has one of the highest in the world, indicative of a strong genetic modifier (20). If we look now at countries outside Europe (and/or North America) we can see that the inverse correlation between poverty and T1D is even more pronounced. Poor sanitation and prevalence of infections seem to protect the inhabitants of developing countries from autoimmune diabetes (see Table 1). A good example is the interdependence between access to clean water, and diseases such as T1D (Figure 1). Indeed many parasitic diseases such as Schistosomiasis require a freshwater environment for transmission. Overall these considerations strongly suggest that the continuous improvement in sanitation and living standards in developed countries is a key factor for the increase of T1D.

**Table 1:** Inverse correlation between Type 1 diabetes (T1D) and hygiene conditions

Country	T1D incidence per thousand, 0–14 yrs	Sanitation (% of households without access to improved sanitation)	H_2_O (% of households with access to improved H_2_O supply)	Low mortality infectious neglected diseases (prevalence)
**Europe**				
Finland	37·4	0	100	Absent
Sweden	28	0	100	Absent
UK	18·9	0	100	Absent
Romania	5	47	58	Absent
Albania	3·6	9	97	Absent
**North America**				
USA	13·8	0	100	Absent
Canada	24·1	0	100	Absent
**Africa**				
Tanzania	0·9	10	68	Endemic
Ghana	No data	28	73	Endemic
**Western Pacific**				
China	0·6	60	75	Frequent
Vietnam	0·3	53	77	Frequent
Singapore	2·5	0	100	Rare/Absent
Australia	17·8	0	100	Rare/Absent
New Zealand	15·2	0	100	Rare/Absent
**East Mediterranean and Middle East**				
Egypt	8	2	97	Rare/Absent
Saudi Arabia	12·3	0	95	Rare/Absent
Kuwait	20·9	No data	No data	Rare/Absent
Yemen	2·5	62	69	Endemic
Afghanistan	1·2	88	13	Frequent
**South/Central America**				
Brazil	8	24	87	Endemic
Argentina	6·4	18	94	Rare/Absent
Venezuela	0·1	32	83	Rare/Absent
Peru	0·4	29	80	Rare/Absent
**South East Asia**				
India	4·2	72	84	Endemic
Nepal	0·6	72	88	Endemic
Bangladesh	4·2	52	97	Endemic

Broadly-speaking there is an inverse relationship between the incidence of T1D and hygiene as gauged by levels of sanitation, access to clean water, and the presence of low mortality infectious diseases (filariasis, leprosy, onchocerciasis, schistosomiasis, soil-transmitted helminths, and trachoma). Adapted from the IDF e-Atlas, http://www.eatlas.idf.org/, © International Diabetes Federation, Brussels.

According to the IDF database the global incidence of T1D in children and adolescents is increasing, with an estimated overall annual rate of about 3%. Before the 1920s childhood diabetes, although uncommon, was rapid and fatal, therefore it could be argued that the introduction of insulin treatment contributed to a subtle increase in the frequency of T1D susceptibility genes. That said, the dramatic rise of T1D in children under 14 years of age in developed countries cannot be explained by genetic factors alone. The T1D epidemic observed over the last 50 years in Western Europe and North America is predicted to plateau. For example, Norway showed no increase over the last decade (21). The high T1D-incidence areas (with the exception of Finland) in Europe appear to have reached a plateau, but the overall trend is still rising in ex-Eastern Bloc countries and in the Middle East, particularly in Kuwait (22–24). Since changes in the environment seem to play a more significant role, predications are that childhood diabetes will not increase exponentially in the high incidence areas but will rather take place in those countries that are gradually seeing an improvement in their living standards and hygiene. For instance, the projections for diabetes incidence in the year 2025 predict a sharp increment in diabetes in the Middle East, South America, Mexico, and South East Asia (see Table 2).

**Table 2:** Diabetes (Type 1 and 2) incidences by region for the year 2003 and projected incidences for the year 2025 (Type 1 and type 2). Ages 20–79

	2003			2025		
		
Region	Population (million)	No. of people with diabetes (million)	Prevalence (%)	Population (million)	No. of people with diabetes (million)	Prevalence (%)
Europe	621	48·4	7·8	646	58·6	9·1
North America	290	23·0	7·9	374	36·2	9·7
Africa	295	7·1	2·4	541	15·0	2·8
Western Pacific	1384	43·0	3·1	1751	75·8	4·3
East Mediterraneanand Middle East	276	19·2	7·0	494	39·4	8·0
South and Central America	252	14·2	5·6	364	26·2	7·2
South East Asia	705	39·3	5·6	1081	81·6	7·5
Total	3823	194	5·1	5251	333	6·3

Adapted from the IDF e-Atlas, http://www.eatlas.idf.org/, © International Diabetes Federation, Brussels.

## ANIMAL MODELS OF HUMAN AUTOIMMUNE DISEASE: THE NOD MOUSE AND *SCHISTOSOMA MANSONI* INFECTION

**Figure 2:**
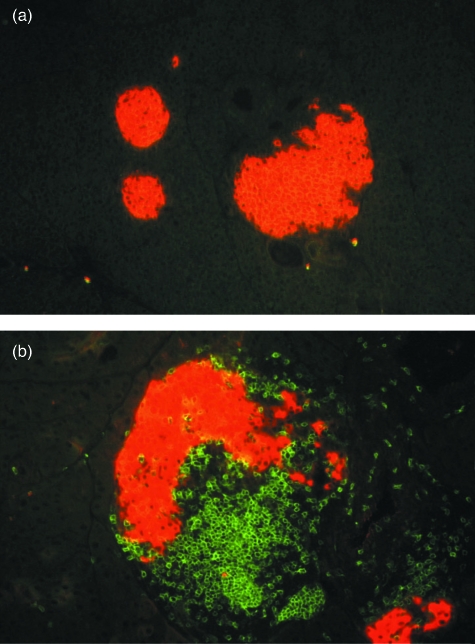
Immunofluorescent staining of a NOD pancreas showing mononuclear cell infiltration. Section of NOD pancreas showing insulin producing β cell mass (orange) and mononuclear cells (green) stained with CD3. Pancreas from young mice show no infiltrate whereas (a) older mice show a spontaneous infiltration around the islet (b) initiating β cell destruction.

Since the 1970s the NOD (Non-Obese Diabetic) mouse has provided a good model for the study of T1D. Initially generated in Japan by Makino and co-workers, the NOD mouse has became one of the most popular models to study T1D (25). NOD mice spontaneously develop T1D, with features similar to the human disease. NOD T1D is under polygenic control and, much like the human disease, associates with particular Class II major histocompatibility (MHC) polymorphisms (26). The pancreas of NOD mice become infiltrated with mononuclear cells around 6 weeks of age, with cells appearing chiefly around the islets and pancreatic ducts. By 8–12 weeks of age the infiltrate progresses to the islets, causing destruction of the β cell mass. Pathology is primarily cell-mediated, with dendritic cells (DC), macrophages (MΦ) and B cells responsible for the initiation of the autoimmune process by presentation of pancreatic antigen and secretion of inflammatory mediators (27,28). Subsequently, CD8+ and CD4+ T cells enter the pancreas, infiltrate the islet area and β cell destruction arises through a Th1-mediated immune response (29) (Figure 2). After 12 weeks of age the clinical signs of disease start to manifest with polydypsia and glycosuria and by the age of 30 weeks 80–100% of female mice are diabetic. NOD mice show a distinct gender difference with female NOD mice developing T1D at a much higher incidence than the males (10–20%). There are variations between colonies and the conditions under which NOD mice are kept appear to greatly influence the rate and frequency of onset of diabetes. It rapidly became clear that NOD mice kept under germ-free conditions developed diabetes at a much faster rate and higher incidence than mice kept under conventional conditions (30). This observation, made independently in many different laboratories, provoked immunologists to consider the possibility that infection and/or exposure to microbial products was responsible for the reduction of T1D incidence in some animal colonies. Experiments designed to test this hypothesis and elucidate the mechanisms of T1D prevention, revealed that infections triggering both Th1- and Th2-like responses could delay or abolish autoimmune pathology in NOD mice (see Table 3).

**Table 3:** Infectious agents or their products that prevent T1D in NOD mice

Agent or product	Th1/Th1 bias	Reference
*Schistosoma mansoni*	Th2	(31)
*Schistosoma mansoni* eggs	Th2	(32)
*Schistosoma mansoni* soluble worm antigen (SWA)	Th2	(32)
*Schistosoma mansoni* soluble egg antigen (SEA)	Th2	(32)
*Heligmosomoides polygyrus*	Th2	C. Lawrence, unpublished data
*Trichinella spiralis*	Th2	C. Lawrence, unpublished data
*Mycobacterium bovis*	Th1	(33)
*Mycobacterium avium*	Th1	(34)
*Salmonella typhimurium*	Th1	(35)
Mouse hepatitis virus (MHV)	Th1	(36)

The NOD mouse appears to be a good model for testing the predictions of the Hygiene Hypothesis, therefore we have used it to study the effects of bacterial and helminth infection on the onset of T1D. *Schistosoma mansoni* infection, or even exposure to antigens derived from this helminth, results in long-lasting prevention of diabetes characterized by a strong Th2 response (31,32). *S. mansoni* protection appears to stem largely from a shift to a non-pathological Th2 response, although there is also evidence for the generation of immunosuppressive regulatory cells (Treg) (32). Essentially similar results have been observed using two other species of helminth, *Heligmosomoides polygyrus* and *Trichinella spiralis* (C. Lawrence, unpublished data). Similarly, infection of NOD mice with live attenuated *Salmonella* bacteria induces a long-lasting protection from T1D (35). Prevention of a Th1-mediated autoimmune disease such as T1D by infection with a classic Th1-stimulating pathogen appears rather paradoxical. Potentially a generalized *Salmonella*-induced IFN-γ release may mediate suppression through its effects on the innate immune system, particularly DC, but the mechanism awaits full characterization (our unpublished observations). At any rate, the invocation of a simple Th1 to Th2 shift is unable to explain all the immunomodulatory effects of microbial infection that lead to prevention of T1D, and may instead require a more complex paradigm incorporating, for example, the action of Treg.

## HELMINTH MODULATION OF THE IMMUNE SYSTEM

**Figure 3:**
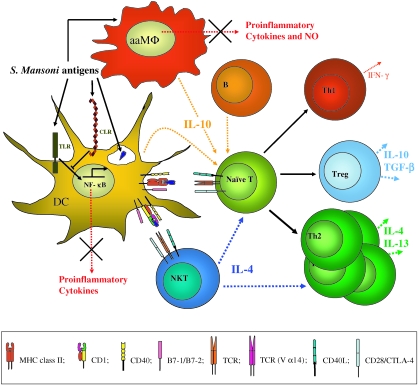
*S. mansoni* modulation of the immune response. *S. mansoni* live helminth and antigens modify cells of the innate immune system through interaction with TLRs and CLRs arresting the production inflammatory mediators and eliciting instead, the release of immunoregulatory cytokines such as IL-10. This results in the generation of suppressive Treg and a bias towards a Th2 response. aaMΦ; alternately activated macrophage.

Amongst the various infectious agents, helminth parasites are regarded as master manipulators of the host immune system, often inducing a long-lasting asymptomatic form of infection (37,38). Parasitic worms can establish and reproduce in mammalian hosts, switching off the inflammatory immune response and inducing a tolerant response to parasite antigens. Following encounter with *S. mansoni* antigens, profound changes are observed in the innate immune system of the host, including modification of DC, MΦ, and NKT cells, phenotype and cytokine secretion (39,40). *S. mansoni* antigens can induce the secretion of regulatory cytokines from these cells as well as B1 B cells (41), resulting in the expansion of Th2 and Treg populations that might be responsible for maintaining self-tolerance (42–45) (see Figure 3). DC and MΦ are fundamental to directing immune responses along either a tolerating or activating pathway, therefore it is not surprising that helminths have evolved strategies targeting receptors on these cells. Toll like receptors (TLRs) and C-type lectin receptors (CLRs), broadly expressed on DCs and MΦs, are the main parasite targets for evading immuno-surveillance (46). More specifically, glycosylated molecules (expressed and secreted by *S. mansoni*) bind to the CLR and antagonize a TLR pro-inflammatory pathway (47). Numerous studies have shown that *S. mansoni* products induce IL-10 production by DCs and have a direct anti-inflammatory effect on DCs by controlling TLR ligand-induced DC maturation (48). *S. mansoni* has also been shown to induce alternatively activated MΦ, which secrete small amounts of inflammatory mediators and inhibit T cell proliferation (49).

The influence of helminth products on the innate immune system is not just restricted to DCs. Depending on the nature of the pathogen, NKT cells can direct the immune response in an appropriate direction by secreting a wide variety of pro- and anti-inflammatory cytokines (50). Schistosomes are rich in glycosylated molecules, which heavily decorate their integument or are actively secreted, and glycolipids presented by CD1d (a non-classical MHC molecule) on antigen presenting cells (APCs) may thus be able to activate regulatory NKT cells (51).

One of the most obvious and well-documented responses to *S. mansoni* is the Th2 dominance in the T cell population. Any initial Th1 response to the parasite is quickly redirected to a state of quiescence (52). The cytokine environment is fundamental for this purpose: large amounts of IL-4, IL-5 and IL-13 are secreted from the T cell pool, reinforcing not just T cell polarization, but also the anti-inflammatory loop on DC and MΦ (32). The parasite is also capable of containing the side-effects of such a strong Th2 response, inducing the secretion of IL-10 and TGF-β by other T cell subtypes (53,54). For example, animals and humans infected or exposed to *S. mansoni* antigens do not automatically develop allergies at a higher incidence (see Figure 3). The *de novo* induction and/or the expansion/recruitment of Treg almost certainly underlies the ability of many parasites to both evade a sterilizing immune response and also suppress both Th1 and Th2 arms of the adaptive immune system (55,56).

## THERAPEUTIC APPLICATION OF PARASITE PRODUCTS AND FUTURE PROSPECTS

Helminths are exquisitely adapted to evading and modulating the mammalian immune response; and interestingly similar evasion mechanisms can be shared among distantly related species (see Table 4).

**Table 4:** Helminth infection or helminth products that prevent autoimmunity in animal models

Agent or product	Autoimmune disease	Reference
*Schistosoma mansoni*	Experimental autoimmune encephalomyelitis	(57)
	Graves’ thyroiditis	(58)
*Schistosoma mansoni* eggs	Experimental autoimmune encephalomyelitis	(59)
	Experimental colitis	(60)
*Trichinella spiralis*	Experimental colitis	(61)
*Trichuris suis*	Inflammatory bowel disease	(62)
*Heligmosomoides polygyrus*	Experimental colitis	(63)
ES-62 (*Acanthocheilonema viteae* product)	Collagen-induced arthritis	(64)

Infection with live helminth or challenge with their products (antigens, eggs etc.) can delay or prevent the induction of autoimmune disease in various disease models.

This begs the obvious question of whether this ability can ever be exploited for therapeutic purposes. The growing body of epidemiological and experimental data detailed above strongly suggests that a reduction in helminth infection is linked to rising rates of autoimmunity and atopy. This offers the real possibility that helminths applied in a controllable clinical setting, could relieve inflammatory disease yet minimize the adverse effects of the parasite. Indeed, several models of autoimmune disease have validated the potential of such an approach. Although still a very young field, limited clinical trials have already been carried out assessing the effects of the porcine whipworm, *Trichuris suis*, on IBD (Inflammatory Bowel Disease) (65). Initial pilot studies using oral ingestion of live *T. suis* ova at regular intervals hinted at a beneficial effect on IBD without any overt side-effects (62). Larger trials, including one double-blinded and placebo controlled, revealed effective relief of Crohn's disease in nearly 80% of patients but much more modest effects in the case of Ulcerative Colitis (62,66). It should be noted that the patients enrolled in these studies were refractory to standard interventions, so any beneficial effect should be welcomed, but larger trials with improved clinical scoring would be desirable (67). A similar trial, this time using the human hookworm *Necator americanus*, is also being carried out in Crohn's patients (68). The caveat of a live parasite approach is only too evident; even if the chosen parasite is unable to productively infect the host patient, as is the case with *T. suis*, there may still be some adverse side-effects, particularly when patients are challenged in conjunction with immunosuppressants, or in otherwise immunosuppressed individuals. In particular, parasite-mediated immunomodulation may compromise the anti-tumour responsiveness of the patient (69–71). Far more desirable then would be to mimic the beneficial effects of helminth infection by using non-infective products derived from them. Aside from the safety issues, the use of helminth products or their synthetic analogues may also allow a finer level of immunomodulatory control or even potency. Emerging proteomics data and the steady progress in the *S. mansoni* sequencing project will also surely illuminate the search for novel and efficacious immunomodulatory helminth-derived products (72,73).

One prediction of the Hygiene Hypothesis is that the rising rate of inflammatory disorders is due specifically to a paucity of infection during infancy, which in turn tunes the immune response in subsequent adulthood to a less pathogenic modality. This being the case, therapeutic dosing of a helminth (or products thereof) to relieve fulminant inflammatory disease in an adult may be relatively ineffective. The patient's immune repertoire, both adaptive and innate, has already been shaped by the absence of parasite antigens, and is subject to only relatively minor perturbations. This may explain the incomplete effects of the *T. suis* infections described above. A prolonged treatment regimen, infection with an attenuated host-specific helminth, or exposure during the supposed critical period of infancy may all potentially improve the efficacy of such an approach. In some not too distant futurity, there may come a day when we all take ‘helminth supplements’ along with our Omega 3 fatty acids, vitamins, and whatever else goes to make up a modern balanced diet.
